# Cognitive Deficits Underlying Error Behavior on a Naturalistic Task after Severe Traumatic Brain Injury

**DOI:** 10.3389/fnbeh.2016.00190

**Published:** 2016-10-14

**Authors:** Kathryn Hendry, Tamara Ownsworth, Elizabeth Beadle, Mathilde P. Chevignard, Jennifer Fleming, Janelle Griffin, David H. K. Shum

**Affiliations:** ^1^School of Applied Psychology and Menzies Health Institute Queensland, Griffith UniversityBrisbane, QLD, Australia; ^2^Rehabilitation Department for Children with Acquired Neurological Injury, Centre National de la Recherche Scientifique, Institut National de la Santé et de la Recherche Médicale, Saint Maurice Hospitals, Saint Maurice, Sorbonne Universités, UPMC Université Paris 06, LIB, F-7013 ParisFrance; ^3^Groupe de Recherche Clinique Handicap Cognitif et Réadaptation—UPMC Paris 6France; ^4^Occupational Therapy Department, Princess Alexandra HospitalBrisbane, QLD, Australia; ^5^School of Health and Rehabilitation Sciences, The University of QueenslandBrisbane, QLD, Australia

**Keywords:** traumatic brain injury, error behavior, naturalistic tasks, cognitive functions, neuropsychological assessment

## Abstract

People with severe traumatic brain injury (TBI) often make errors on everyday tasks that compromise their safety and independence. Such errors potentially arise from the breakdown or failure of multiple cognitive processes. This study aimed to investigate cognitive deficits underlying error behavior on a home-based version of the Cooking Task (HBCT) following TBI. Participants included 45 adults (9 females, 36 males) with severe TBI aged 18–64 years (*M* = 37.91, *SD* = 13.43). Participants were administered the HBCT in their home kitchens, with audiovisual recordings taken to enable scoring of total errors and error subtypes (Omissions, Additions, Estimations, Substitutions, Commentary/Questions, Dangerous Behavior, Goal Achievement). Participants also completed a battery of neuropsychological tests, including the Trail Making Test, Hopkins Verbal Learning Test-Revised, Digit Span, Zoo Map test, Modified Stroop Test, and Hayling Sentence Completion Test. After controlling for cooking experience, greater Omissions and Estimation errors, lack of goal achievement, and longer completion time were significantly associated with poorer attention, memory, and executive functioning. These findings indicate that errors on naturalistic tasks arise from deficits in multiple cognitive domains. Assessment of error behavior in a real life setting provides insight into individuals' functional abilities which can guide rehabilitation planning and lifestyle support.

## Introduction

Impairments in error self-regulation are common to many neurological disorders, including traumatic brain injury (TBI) (Hart et al., 1998; O'Keeffe et al., 2007), stroke (Stemmer et al., 2004), and dementia (Giovannetti et al., 2002; Bettcher et al., 2008). These impairments most typically arise from damage or disease processes disrupting the prefrontal cortex and connecting pathways which support the capacity to accurately reflect upon and regulate one's own behavior. Error self-regulation problems are one of the main reasons people experience a loss of independence and vocational difficulties following TBI (Ownsworth et al., 2015). A greater understanding of the cognitive processes underlying error self-regulation is needed to guide rehabilitation interventions.

Error self-regulation is a dynamic process mediated by two interacting yet dissociable components; namely, performance monitoring and regulative control (Kerns et al., 2004; Larson et al., 2007). Performance monitoring or evaluation is mediated by the anterior cingulate cortex (ACC), which in turn signals the dorsolateral prefrontal cortex (DLPFC) to recruit top-down processes (i.e., regulative control) that support behavioral adjustments to avoid or correct an error (Larson et al., 2007). Although error detection is usually accompanied by efforts to correct or avert the error, research indicates that people with TBI can show an awareness of errors (e.g., pausing mid-action or verbalizing) without making appropriate performance adjustments (Hart et al., 1998; Larson et al., 2012).

Following brain injury errors may occur due to impairments in both lower and higher-order cognitive processes such as attention, memory and executive function. Errors can result from a loss of arousal and alertness or “mind wandering” (O'Keeffe et al., 2007; Allan Cheyne et al., 2009). Further, impairments in episodic or prospective memory can lead to a failure to remember instructions or to carry out an action at the right moment. Errors can also be related to a lack of planning and goal-directed behavior which affects “the ability to orchestrate thought and action in accord with internal goals” (Larson et al., 2007, p. 961). Different neurocognitive mechanisms are likely to underlie particular error types; for example, loss of drive and initiation may contribute to errors of omission (Hart et al., 1998), whereas an inability to inhibit a prepotent response contributes to errors of commission or impulsive responses (Rochat et al., 2013).

Approaches to assessing error self-regulation in research and clinical practice vary in the extent to which they resemble the cognitive demands of real world tasks; a concept referred to as “verisimilitude” (Wood and Liossi, 2006). Many neuropsychological tests assess error behavior (e.g., rule breaks, repetitions, perseverations), including the Stroop, Trail Making Test, Tower Test, Wisconsin Card Sort Test, Hayling Test, and fluency tests. Although often possessing strong psychometric properties, traditional neuropsychological tests have been criticized for failing to adequately capture or reflect individuals' performance in real-world settings; that is, to lack ecological validity (Chaytor and Schmitter-Edgecombe, 2003). This has been attributed to the often highly structured nature of tasks (e.g., detailed instructions), which may scaffold participants' behavior to the extent that impairments are concealed (Sohlberg and Mateer, 2001). Further, testing is often conducted in controlled distraction-reduced environments, which are designed to facilitate optimal performance, but are rarely encountered in everyday life (Chaytor and Schmitter-Edgecombe, 2003). Thus, performance on traditional tests of executive function may not always reflect individuals' capacities in the real-world.

Over the past two decades there has been a move toward the development of more ecologically valid neuropsychological tests to assess people's capacities in real-world settings (Hart et al., 1998; Bottari et al., 2006). Test batteries such as the Behavioral Assessment of Dysexecutive Syndrome (BADS; Wilson et al., 1996) and Test of Everyday Attention (Robertson et al., 1996) include subtests designed to simulate real life tasks while maintaining practical utility (e.g., feasible administration time and application in different settings). Other approaches use computerized simulation tasks and virtual reality platforms, such as the Breakfast Task (Tanguay et al., 2014) and Virtual Library Test (VLT: Renison et al., 2012), with programmes designed to resemble everyday contexts. However, evidence of the relationship between performance on such measures and corresponding real-world tasks is mixed. For instance, Renison et al. (2012) reported a significant association between performance on the VLT and a real-life equivalent task. In contrast, Tanguay et al. (2014) found that participants' performance on the Breakfast Task was not significantly related to their ability to prepare an actual meal. Such findings highlight potential limitations of computerized tasks in terms of the capacity to represent the complexities of the real-world, such as the multisensory experiences that accompany cooking (McGuire, 2014).

Observation of performance on real tasks in controlled (e.g., Meal Preparation Scale; Jongbloed et al., 1988) or naturalistic environments (e.g., Multiple Errands Test, MET; Shallice and Burgess, 1991) have been recommended as part of a comprehensive neuropsychological assessment. The Cooking Task (CT) is a naturalistic assessment of error behavior designed by Chevignard et al. (2000) to have good ecological validity and sensitivity to impairments after TBI. As a multi-tasking cooking activity undertaken in a hospital kitchen, participants are required to prepare a chocolate cake and an omelet using cooking implements and ingredients supplied by the examiner. Performance is evaluated primarily by the type and number of errors made during task completion. Specifically, scoring of the CT involves recording the total number of errors and error types, which include: Omissions, Additions, Sequence/Substitutions, Estimation errors, and Commentary/Questions. Three additional indices assess the presence of Dangerous Behavior (Yes/No), Goal Achievement (Yes/No) and task duration (minutes).

The CT has demonstrated good psychometric properties, including inter-rater reliability, and evidence of discriminative validity; that is, the ability to differentiate between the performance of people with brain injury and healthy controls (Chevignard et al., 2000, 2008; Poncet et al., 2015). Variance in total CT errors was significantly explained by performance on tests of executive function, including the Six Elements Test (SET; ~26%), Wisconsin Card Sort Test (WCST; ~10%), Verbal Fluency (~6.5%) and Brown-Peterson Paradigm (~6.7%) (*p* < 0.05). Performance on the SET also predicted Goal Achievement (~25%) and the presence of Dangerous Behavior (~40%). A significant association was found between CT total errors and dysexecutive symptoms (*r* = 0.57). Females, younger participants, and those with greater cooking experience were found to perform better on the CT (Chevignard et al., 2008).

Performance on complex functional tasks such as the CT is likely to vary according to individuals' premorbid abilities, the neuropathology of injury (i.e., nature and severity of injury), and the environmental context (Bottari et al., 2006; Ponsford, 2013). Task dimensions such as novelty, structure, and familiarity of the environmental context are likely to influence the degree to which errors occur during performance (Shallice and Burgess, 1991; Bottari et al., 2006; Chan et al., 2008). Although the CT is useful for assessing people's functional capacity within a hospital setting, assessment of individuals' error behavior in their own homes potentially provides greater insight into their real-world functioning (e.g., use of compensatory strategies and relative strengths and limitations). Accordingly, we developed a home-based version of the Cooking Task (HBCT) for administration in participants' home-kitchens (Ownsworth et al., 2015). The HBCT utilizes the same instructions as the CT and uniform materials (utensils and ingredients) brought by the researcher. Consistent with the CT, the HBCT has demonstrates sound inter-rater reliability (total errors ICC = 0.96) and evidence of discriminative validity (Ownsworth et al., 2015; see Methods).

Although performance on the hospital version of the CT was closely related to tests of executive function (Chevignard et al., 2008), the familiarity inherent in participants' home kitchens may alter the cognitive demands of the task, and hence implicate alternative cognitive processes. Further to this, variability in the presence of distractors and environmental triggers for habitual behavior in people's home kitchens may modify other task dimensions such as task difficulty and novelty, and lead to more naturalistic behaviors (e.g., talking to the examiner), or strategy use. As such, errors occurring on the HBCT may more closely approximate error behavior in everyday life than those on the CT.

The present study aimed to investigate cognitive deficits related to error behavior on the HBCT following severe TBI. It was hypothesized that greater errors on the HBCT would be significantly related to poorer cognitive status (i.e., attention, memory, and executive functioning) after controlling for relevant covariates (e.g., age, gender, and cooking experience). The findings were expected to provide theoretical insights into cognitive processes supporting error self-regulation and guide clinical interventions for individuals with severe TBI.

## Methods

### Participants

Participants with severe TBI were recruited from three metropolitan brain injury rehabilitation services in Brisbane and Sydney. They were eligible for the study if they were aged 18–65 years, had experienced a severe TBI [based on duration of posttraumatic amnesia (PTA) and Glasgow Coma Scale (GCS) score], were medically stable, and lived within a 50 km radius of Brisbane and Sydney metropolitan centers. Individuals were excluded if they were unable to provide informed consent, displayed a combination of severe behavioral, motor, perceptual, language, or cognitive impairments which precluded participation in research of this nature, or exhibited psychotic or severe mood symptoms not effectively managed. All participants were deemed to have sufficient English language proficiency to understand the study material and instructions. Eligible individuals were initially approached by treating clinicians and invited to participate. A total of 69 individuals with severe TBI were approached. Of these, 24 did not participate for the following reasons: were not eligible (*n* = 12), declined (*n* = 6) or were unable to be contacted following initial verbal consent (*n* = 6). Demographic and injury characteristics of the TBI sample are summarized in Table 1. As shown, the 45 participants (80% male) were aged between 18 and 64 years and their time since injury ranged from 4 to 160 months (*M* = 31.6 months, *SD* = 36.9).

**Table 1:** **Participants' Demographic and Medical Characteristics (*n* = 45)**.

**Characteristics**	**TBI Total Group *M*(*SD*), range/*N* (%)**
Age (years)	37.91 (13.43) 18 – 64
**GENDER**	
Male	36 (80)
Female	9 (20)
**PREMORBID COOKING EXPERIENCE**	
Rarely	16 (35.60)
Frequent, but not baking	21 (46.7)
Frequent and bakes	8 (17.8)
Years of Education	12.64 (2.39) 8 –18
**LIVING STATUS**	
Alone	8 (17.8)
With flatmate or other residents	4 (8.9)
With Family	17 (37.08)
With Spouse	16 (35.6)
**RELATIONSHIP STATUS**	
No Spouse	28 (62.2)
Spouse	17 (37.8)
**CAUSE OF INJURY**	
Traffic Accident	21 (46.7)
Assault	5 (11.1)
Fall	16 (35.6)
Sport	2 (4.4)
Falling Object	1 (2.2)
Time Since Injury (months)	31.58 (36.92) 4 –160
TBI Severity (GCS)^a^	5.51 (3.04) 3 –13

a *Although some participants had a GCS of >8 recorded, data on duration of PTA confirmed that they had sustained a severe TBI*.

### Measures

#### Home-based cooking task (HBCT)

The HBCT is a home-based version of the CT (Chevignard et al., 2008), a naturalistic assessment of executive functions. The CT has been found to have good psychometric properties, including inter-rater reliability [CT Total errors: *ICC* = 0.93; error subtypes: *ICC* = 0.65 (Omissions) to 0.95 (Commentary/Questions)], and evidence of discriminative, convergent and concurrent validity (Chevignard et al., 2000, 2008; Poncet et al., 2015). Participants completed the HBCT in their home kitchens according to standardized CT procedures; the same instructions, recipe, implements, and scoring as the CT were adopted. A French to English language translation of the CT manual and materials was used (Taillefer et al., 2013), and the researchers were trained in person by one of the test developers (author MPC). Participants were given verbal instructions regarding the task goals (i.e., to make a chocolate cake and an omelet for two people) and task rules; namely, to only use the implements and ingredients provided by the assessor, to act as if they are alone, and to advise when the task is completed. Participants were provided with a written copy of the instructions to refer to throughout the task. Prior to commencing the HBCT, participants reviewed the instructions, and repeated what they were required to do, to confirm their understanding.

Participants were advised that the recipe for the chocolate cake was one of the recipes contained in a folder (holding 5 recipes). In accordance with CT guidelines, no recipe was provided for the omelet. Due to differing food practices in Australia, participants were given the option of making scrambled eggs if unsure how to make an omelet. This was discussed and decided prior to the participant commencing the task. Standard ingredients and implements necessary to make the chocolate cake and omelet, as well as distractor items (e.g., packet of raisins, cheese grater) were provided and arranged as uniformly as possible within the layout of participants' home kitchens. The HBCT is scored according to five error types (Omissions, Additions, Sequence/Substitutions, Estimation errors, and Commentary/Questions), from which a total error score was derived by summing the number of errors scored across all five continuous indices. Additionally, two dichotomous indices were recorded; the presence or absence of Dangerous Behavior (Y/N); and Goal Achievement (Y/N). The time taken by participants to complete the task (in minutes) was also recorded. Table 2 provides a description and examples of errors corresponding to the HBCT indices.

**Table 2:** **Home-Based Cooking Task Descriptive Error Classification Indices (Source: CT Manual; Taillefer et al., 2013)**.

**Error**	**Description**	**Examples of Errors Made by TBI Participants**
Omission	Any action or sequence of actions normally required to reach the goal, which is either omitted or incompletely performed.	■ Omitting an ingredient (e.g., forgetting to add flour to the cake batter) or an instruction stated in the recipe (e.g., not mixing the batter between ingredients)
Addition	Any action or sequence of actions unnecessary to the completion of the task. Such actions may be part of the task or totally irrelevant to the task.	■ Using ingredients not required for the recipe or moving or touching an object without any reason or apparent goal (e.g., picking up the sugar bag then putting it down again without having used it
Commentary/Question	Any comment, question, joke or request for help made by the participant after the task has commenced	■ Examples: “Where is the sugar?,” “Would you like some omelet/eggs?,” “How do I turn on the oven?”
Sequence/Substitution	Incorrect object selection or use, or use of an object inappropriate to the goal. Any sequence error.	■ Not following the order in the recipe when adding ingredients or beating up the eggs with a tablespoon instead of using a whisk or fork
Estimation	Errors related to poor estimation of quantity, size, time, or temperature etc.	■ Adding the incorrect amount of an ingredient or leaving the cake to cook in the oven for too long
Goal Achievement	Did the participant complete task? Scoring: 0 = task complete; 1 = task incomplete.	Circumstances/actions resulting in a failure to complete the task include: ■ Omitting a required ingredient from the cake ■ Overall time to complete task exceeding 2 h ■ Commencing the wrong cake recipe without self-correcting (requiring prompt) ■ Not initiating making the omelet (requiring prompt) ■ Forgetting to remove cake from oven
Dangerous Behavior^a^	Was any behavior/action or inaction by the participant potentially dangerous or harmful? Scoring: 0 = no dangerous behavior observed; 1 = At least one dangerous behavior observed.	Examples of Dangerous Behavior include: ■ Removing the hot cake tin from oven with bare hands ■ Placing a metal bowl in the microwave ■ Leaving the oven door open with oven left on

a *the researcher intervened immediately to ensure the participant's safety*.

Previous pilot research (Ownsworth et al., 2015) identified that inter-rater reliability for scoring the HBCT ranged from good to excellent for continuous error indices (ICCs = 0.63–0.96), and fair to excellent for dichotomous error indices (*k* = 0.57–0.86). Further, the HBCT was found to distinguish between the performance of participants with severe TBI and controls matched on age, gender, and cooking experience. TBI participants made significantly more total errors and errors of each subtype than controls, and were more likely to exhibit dangerous behavior (29%) than controls (0%).

#### Digit span

The Digit Span test is a well validated subtest from the Wechsler Adult Intelligence Scale III (Wechsler, 1997) that assesses auditory attention and working memory. This test consists of two parts: Digits Forward and Digits Backward. In Digits Forward, the participant is required to repeat after the examiner a series of numbers in the exact order. Conversely, in Digits Backward the participant repeats the number sequence in reverse order. This study utilized an age-based scaled score derived from the total raw score.

#### Hopkins verbal learning test-revised

The Hopkins Verbal Learning Test-Revised (HVLT-R; Brandt and Benedict, 2001) is a standardized measure of verbal learning and memory. The examiner reads aloud a list of 12 semantically related words (e.g., gem stones, animals). The word list is repeated twice (a total of three trials) and participants are asked to recall the list immediately after each trial. After a 20–25 min delay participants are asked to recall the list from memory. Standardized scores are derived using age-based normative data. For this study, two indices were used; namely, total immediate recall (verbal learning), and delayed recall.

#### Modified stroop test

The Modified Stroop Test (Strauss et al., 2006), also known as the Victoria Stroop Test, is a measure of selective attention, set shifting, and inhibitory control. It involves three trials: Trial 1 is color naming; Trial 2 is color ink naming of words; Trial 3 is color naming when the words are printed in non-matching ink color. The individual is required to name the colors as quickly as possible without making mistakes. In order to control for processing speed, an interference control score was calculated by dividing the time required to name colors in the control task (Trial 1) from the time required to name the colors in the interference task (Trial 3). This score was converted to a standardized score based on age-based norms (Strauss et al., 2006).

#### Zoo map test

A subtest of the BADS, the Zoo Map Test (Wilson et al., 1998) assesses planning, problem-solving, and self-regulation (Norris and Tate, 2000). Part 1 requires participants to use a map to formulate and implement a plan to navigate a fictional zoo to visit particular animals, while following specific rules (e.g., only use certain sections of the path once), and finish at the picnic area. In Part 2, participants are provided with a plan they must follow to navigate the zoo successfully (essentially a solution to Part 1); involving basic sequencing skills as the generation of a plan is not required. Total raw scores are derived from points awarded for locations visited in the correct order, with points deducted for rule violations (maximum score 16). Raw scores for both Parts are converted to a profile score ranging from 0 to 4 with higher scores indicating better executive functioning. Points are deducted from the overall profile score for time violations. The number of errors made in Part 1 (rule following/violations) was also used in the analysis due to the focus on error behavior.

#### Hayling sentence completion test (hayling)

The Hayling (Burgess and Shallice, 1997) assesses response initiation and suppression in accordance with Norman and Shallice (1986) model. In Part 1 (15 sentences), participants are asked to generate an appropriate word to complete each sentence as quickly as possible, with response speed recorded. In Part 2 (15 sentences), participants are asked to provide a word that makes no sense in regards to the sentence (i.e., inconsistent with a pre-potent response), again as quickly as possible. Four scaled scores are derived from the time taken to complete Parts 1 and 2 (response initiation), number of errors on Part 2 (response suppression), and the sum of these three scaled scores (i.e., overall score). As used in the present study, the final overall scaled score ranges from 1 (impaired) to 10 (very superior); a score of 6 indicates average ability (Burgess and Shallice, 1997).

#### Trail making test (TMT)

The TMT (Partington and Leiter, 1949) is a measure of visual attention, processing speed and mental flexibility. A pencil and paper test, TMT consists of two parts: Trails A and B. Trails A requires the participant to draw a continuous line that connects circled numbers in correct order. In Trails B, participants draw a line that alternates between numbers and letters in ascending/advancing order (e.g., 1-a-2-b-3…). Time taken (in seconds) to complete each part is recorded. To isolate the higher-order cognitive demands of divided attention and cognitive flexibility, a difference score (Trails B-A) was used along with Trails A in the analysis. Raw scores were converted to standardized scores using age norms and reflected, so that higher positive *z*-scores represented better visual attention and mental flexibility (Strauss et al., 2006).

### Procedure

Ethical clearance for this study was granted by hospital and university human research ethics committees. Following informed consent procedures, data were collected during a face-to-face assessment session in participants' own homes. Demographic and health information was collected first (note: medical data were accessed from hospital records), followed by administration of the HBCT. Administration of the HBCT was video-recorded to allow for later scoring. Administration of the HBCT and the neuropsychological tests was typically conducted over two sessions to minimize fatigue. Although family members were often present in the home during data collection, they were asked to remain in another room to minimize distractions.

## Results

### Data analysis

The Statistical Package for Social Sciences (SPSS) Version 22 for Windows was utilized for all analyses. Data were screened for errors, missing values and relevant statistical test assumptions. Nine variables were identified with problematic skew/kurtosis and/or univariate outliers (GCS, Trails A, Trails B-A, Zoo Map Part 1 errors, and HBCT Total Errors, Additions, Estimation errors, Commentary/Questions and completion time). Analyses were conducted using transformed and untransformed variables; results for transformed variables are reported where they altered significance (note: this was the case for Zoo Map Part 1, Estimation errors, Commentary/Questions and HBCT completion time).

### Descriptive data

Descriptive statistics on all neuropsychological tests for the TBI sample (*n* = 45) are presented in Table 3. As shown, based on mean standardized scores, TBI participants typically performed in the impaired range on each test. On the HBCT, participants took on average 55 min to complete the task (range: 36–119 min), and displayed a mean total error score of 50.27 (*SD* = 27.79). Additions were the most common error type (*M* = 21.36, *SD* = 13.49), whereas Estimation errors were least common (*M* = 4.07, *SD* = 3.53). Nearly half (44.4%) of the sample committed a dangerous behavior, and 51.1% failed to achieve the goal or complete the task successfully.

**Table 3:** **Descriptive Data for the HBCT Error Indices and Neuropsychological Tests (*n* = 45)**.

**Test Index**	***M***	***SD***	**Range**
HBCT Total Errors	50.27	27.79	17 – 134
Omissions	4.27	3.33	0 – 12
Additions	21.36	13.49	1 – 64
Sequence/Substitution	6.31	4.50	0 – 17
Estimation	4.07	3.53	0 – 14
Commentary/Questions	14.09	15.81	0 – 76
HBCT Dichotomous Variables	Yes *n* (%)		
Goal Achievement^a^	23 (51.1)		
Dangerous Behavior^b^	20 (44.4)		
Trails A (*z*-score for seconds)	−3.16	3.80	−14.74 – 1.78
Trails B-A (*z*-score)	−4.53	5.60	−25.51 – 1.50
Zoo Map Total Profile Score	1.18	0.94	0 – 4
Zoo Map Version 1 (errors)	3.47	3.41	0 – 13
Hayling Overall Profile Score	3.93	2.22	1 – 10
HVLT Immediate	−2.88	1.33	−5.47 – 0.32
HVLT Delayed	−3.11	1.73	−6.06 – 0.11
Digit Span	8.58	2.86	1 – 14
Modified Stroop Test	−1.21	1.43	−4.80 – 1.13

HBCT = Home-Based Cooking Task;

a Goal Achievement: Yes = task complete (no error);

b *Dangerous behavior: Yes = committed a dangerous behavior (error)*.

### Cognitive deficits related to error behavior on the HBCT

Pearson product-moment correlations were conducted to assess the associations between the HBCT error indices and cognitive test performance. Correlations between the indices of the HBCT indicated that a greater number of Additions was associated with more Sequence/Substitution errors (*r* = 0.52, *p* < 0.01), increased Commentary/Questions (*r* = 0.39, *p* < 0.01) and longer completion time (*r* = 0.51, *p* < 0.001). Further, greater Sequence/Substitution errors were related to longer completion time (*r* = 0.37, *p* < 0.05), and Omissions were associated with the presence of Dangerous Behaviors (*r* = 0.31, *p* < 0.05). There were no other significant correlations between error types.

As shown in Table 4, there were no significant correlations between the neuropsychological tests and HBCT Total Errors, Additions, Sequence/Substitutions, or Dangerous Behavior (*p* > 0.05). Significant moderate correlations were found between Omissions and Digit Span (*r* = −0.37, *p* < 0.05), Trails A (*r* = −0.41, *p* < 0.01), Trails B-A (*r* = −0.31, *p* < 0.05), Zoo Map Part 1 errors (*r* = 0.31, *p* < 0.05), Hayling (*r* = −0.43, *p* < 0.01), HVLT-R immediate (*r* = −0.48, *p* < 0.01) and delayed recall (*r* = −0.42, *p* < 0.01). Such findings indicated that the tendency to omit aspects of the task (e.g., steps or ingredients) was associated with poorer auditory and visual attention, cognitive flexibility, rule following, response initiation and suppression, new verbal learning, and delayed recall.

**Table 4:** **Correlations between the Home-Based Cooking Task and Neuropsychological Tests (*n* = 45)**.

	**Trails A^a^**	**Trails B-A^b^**	**Zoo Map Profile Score**	**Zoo Map Part 1 (errors)**	**Hayling**	**HVLT Immediate**	**HVLT Delayed**	**Digit Span**	**Stroop Color**
HBCT Total Errors	−0.03	−0.08	−0.06	0.04	0.06	−0.08	−0.21	0.10	−0.05
Omissions	−0.41^**^	−0.31^*^	−0.05	0.31^*^	−0.43^**^	−0.48^**^	−0.42^**^	−0.37^*^	−0.22
Additions	0.01	0.14	−0.04	−0.27	0.21	0.06	−0.05	0.19	−0.05
Sequence/Substitutions	0.05	0.22	−0.17	−0.18	0.12	−0.07	−0.12	0.15	−0.02
Estimations	−0.28	−0.09	−0.27	0.34^*^	−0.37^*^	−0.38^**^	−0.50^**^	−0.32^*^	−0.43^**^
Commentary/Questions	0.03	0.00	−0.05	0.32^*^	−0.07	−0.06	−0.15	0.03	0.13
Goal Achievement^a^	−0.06	0.05	−0.14	0.04	−0.29^*^	−0.27	−0.37^*^	−0.22	−0.33^*^
Dangerous Behavior^b^	−0.22	−0.03	−0.08	0.11	−0.08	−0.18	−0.21	−0.07	−0.20
Completion Time	0.24	0.34^*^	0.28	−0.16	0.26	0.23	0.37^*^	0.25	0.07

* *p < 0.05*,

** *p < 0.01, (two-tailed)*.

a *Goal Achievement: 0 = task complete; 1 = task not completed*.

b *Dangerous behavior: 0 = no dangerous behavior; 1 = dangerous behavior*.

Significant moderate to large correlations were found between Estimation errors and Digit Span (*r* = −0.32, *p* < 0.05), Hayling (*r* = −0.37, *p* < 0.05), Zoo Map part 1 errors (*r* = 0.34, *p* < 0.05), HVLT-R immediate memory (*r* = −0.38, *p* < 0.01) and delayed memory (*r* = −0.50, *p* < 0.001), and Modified Stroop (*r* = −0.43, *p* < 0.01). Hence, individuals making more Estimation errors (e.g., misjudging quantity, size and time) had greater difficulty with auditory attention/working memory, rule following, response inhibition, and suppression, new verbal learning and delayed recall, and interference control.

Zoo Map Part 1 errors was the only neuropsychological index significantly correlated with Commentary/Questions (*r* = 0.32, *p* < 0.05), indicating that participants who made more comments or asked more questions also had more rule violations on a measure of planning and self-regulation. Participants who successfully completed the HBCT (i.e., Goal Achievement) had better response initiation and suppression (Hayling; *r* = −0.29, *p* < 0.05), delayed memory (HVLT-R; *r* = −0.37, *p* < 0.05), and interference control (Modified Stroop; *r* = −0.33, *p* < 0.05). Participants taking longer to complete the HBCT had poorer cognitive flexibility (Trails B-A; *r* = −0.34, *p* < 0.05), and delayed memory (HVLT-R; *r* = 0.37, *p* < 0.05).

Due to the multiple neuropsychological indices related to HBCT Omissions, Estimation errors, Goal Achievement and completion time, regression analyses (i.e., linear and binary logistic regression) were conducted to determine whether neuropsychological test performance was related to performance on the HBCT after controlling for potential covariates. Time since injury, injury severity, relationship status, education, and estimated premorbid intelligence were not significantly correlated with any of these HBCT indices (*p* > 0.05). However, older participants made fewer Omissions and Estimation errors (*p* < 0.05). Further, females made fewer Omissions (*p* < 0.01), and were more likely to complete the HBCT (*p* < 0.05) than males. Those with greater prior cooking experience made significantly fewer Omissions (*p* < 0.001) and Estimation errors (*p* < 0.05), were more likely to complete the HBCT (*p* < 0.05), and took less time to complete the task (*p* < 0.01).

To maintain an adequate participant to variable ratio (i.e., 10:1), where numerous neuropsychological indices were correlated with a HBCT index, only those more strongly associated (i.e., *p* < 0.01) were included as independent variables, and neuropsychological indices from the same test (e.g., immediate and delayed memory on the HVLT-R) were not entered in the same regression model. Partial correlations revealed that age and gender were not significantly related to performance on the HBCT when controlling for cooking experience. Therefore, cooking experience was entered in step 1 of all regression models.

#### Cognitive tests related to HBCT omissions

In step 1 of the regression model, cooking experience significantly accounted for 25% of variance in Omissions, *R*^2^ = 0.25, adjusted *R*^2^ = 0.23, *F* = 13.94, *p* = 0.001. In step 2, Trails A, HVLT-R immediate memory and Hayling significantly accounted for 16% additional variance, *R*^2^ = 0.40, adjusted *R*^2^ = 0.34, *F* = 6.72, *p* < 0.001). None of the neuropsychological tests accounted for significant unique variance in the model (*p* > 0.05). However, cooking experience contributed significant unique variance to Omissions (β = −0.31, *sr*^*2*^ = −0.08, *p* = 0.03).

#### Cognitive tests related to HBCT estimation errors

Cooking experience significantly accounted for 15% of the variance in Estimation errors in step 1, *R*^2^ = 0.15, adjusted *R*^2^ = 0.13, *F* = 7.44, *p* = 0.009. In step 2, HVLT-R delayed memory and Modified Stroop explained an additional 16% of the variance in Estimation errors, (*R*^2^ = 0.31, adjusted *R*^2^ = 0.26; *F* = 5.91, *p* = 0.02). No variable explained significant unique variance in Estimation errors (*p* > 0.05).

#### Cognitive tests related to goal achievement on the HBCT

In Step 1 of the logistic regression, cooking experience reliably distinguished between those who achieved the goal and those who failed to achieve the goal, Nagelkerke's *R*^2^ = 0.17, χ^2^ = 5.62, *p* = 0. 018. The addition of Modified Stroop, Hayling, and HVLT-R delayed memory in Step 2, improved prediction of Goal Achievement, Nagelkerke's *R*^2^ = 0.32, χ^2^ = 11.11, *p* = 0. 011. Prediction success overall was 70% (80% for complete and 62% for incomplete). The Wald criterion indicated that none of the variables were significant unique predictors of Goal Achievement (*p* > 0.05, *Exp(B)* < 1).

#### Cognitive tests related to completion time on the HBCT

In Step 1, cooking experience accounted for significant variance (15%) in time taken to complete the HBCT, *R*^2^ = 0.15, *F* = 7.40, *p* = 0.01. After entering Trails B-A and HVLT-R delayed memory in Step 2, the variance in completion time explained increased to 22% (*R*^2^ = 0.22, *F* = 3.86, *p* = 0.02). No cognitive test explained significant unique variance in completion time (*p* > 0.05).

## Discussion

Assessment of error behavior during real life tasks in the home environment can help to determine the safety, independence and supervision needs of individuals who have sustained brain-injury (Bottari et al., 2006; Chevignard et al., 2008). This study investigated cognitive deficits related to error behavior on a HBCT following severe TBI. After controlling for prior cooking experience, poorer attention, memory and executive functions were significantly associated with greater Omissions and Estimation errors, lack of goal achievement, and longer completion time.

Numerous cognitive processes were found to be associated with performance on the HBCT. However, this was not the case for all error types or the total error score. Specifically, there were no significant associations between the neuropsychological tests and HBCT total errors, Additions, Sequence/Substitution errors and Dangerous Behavior. Therefore, variations in performance across these indices do not appear to be influenced by participants' cognitive functioning, as assessed by the tests selected in this study. It was also noteworthy that other variables such as prior cooking experience, time since injury and injury severity were not related to these indices. It is possible that variations in the environment (e.g., kitchen layout, presence of distractors) or client-related factors (e.g., fatigue, anxiety) influenced these indices.

Zoo Map Part 1 errors were significantly related to Commentary/Questions, thus indicating that behavioral self-regulation (i.e., rule following) influences people's tendency to make a comment or joke, or ask a question to request help, despite being asked to act as if they are alone. Other HBCT error indices were associated with multiple cognitive processes. In particular, greater Omissions were associated with reduced attention and working memory, verbal learning and delayed recall, cognitive flexibility, and greater difficulty initiating and suppressing responses. Such errors relate to the omission or incomplete performance of an action or sequence of actions required to complete the task. Thus, various cognitive processes are engaged to support individuals to monitor their own performance and carry out the task instructions.

Estimation errors were related to similar aspects of cognitive functioning to Omissions; however, interference control (Modified Stroop) was more strongly associated with these errors than other aspects of executive functioning. Such findings indicate that poor estimation of quantity, size, time, and temperature while cooking is related to a difficulty in preventing competing internal or external stimuli from disrupting goal-directed behavior. The inability to complete the HBCT successfully and achieve the goal due to “fatal” errors (e.g., commencing the wrong recipe, needing a prompt to prepare the omelet) was also related to poorer interference control, initiation, and suppression of responses, and delayed memory. Participants taking longer to complete the HBCT had poorer cognitive flexibility and delayed memory.

A key difference between the findings of the present study and those of Chevignard et al. (2008) is that, in their previous study, total errors on the CT was related to indices of executive function, including multi-tasking (SET), behavioral self-regulation (WCST perseverative errors), semantic fluency and working memory. Their findings indicate that various higher-order cognitive functions are engaged to support effective performance on the CT, and thus influence global level of errors. A potential explanation for these contrasting results relate to the different neuropsychological measures administered, with the current study employing briefer tests of executive function alongside tests of attention and memory. However, there were also variations in the settings in which the task was administered (hospital vs. home), and sampling differences with respect to etiology and time since injury. All participants in the present study had severe TBI and were living in the community, whereas participants in the study by Chevignard et al. (2008) were receiving inpatient care and had sustained ABI from mixed causes including stroke.

Notwithstanding these methodological differences, the greater familiarity (or lack of novelty) of the task setting for the HBCT relative to the CT is considered a key factor accounting for the differences in the findings with respect to cognitive processes related to total errors. Although not possible to quantify, variations in participants' kitchens (e.g., bench space, ovens, and presence of distractions) may have influenced total errors on the HBCT to a greater extent than participants' cognitive abilities. Further, the presence of triggers for habitual behavior (e.g., using one's own ingredients and utensils, having a conversation while cooking) in the home kitchen may have contributed to errors that are less likely to occur in an unfamiliar or novel setting. Therefore, despite using the same task instructions and equipment, the setting for administration appears to influence the cognitive abilities implicated in performance and the extent and nature of errors (Bottari et al., 2006).

Some study limitations are important to acknowledge. First, the sample size was modest for regression analysis and the study may have lacked statistical power to determine the shared and unique contribution of neuropsychological tests to HBCT performance. Second, it was not feasible to directly compare performance on the CT and HBCT, or investigate cognitive processes influencing performance on each version. Therefore, only general comparisons could be made across studies, which employed different samples and neuropsychological tests. Some caution is therefore needed in drawing conclusions about differences between these tasks. Third, the assessment of cognitive functions focused on attention, memory and executive function domains. Impairments in language and visuo-spatial function are also likely to contribute to errors for people with TBI, but were not assessed in this study (note: individuals with severe aphasia and/or perceptual impairments were excluded). Bearing in mind these limitations, the present findings have both theoretical and clinical implications.

After controlling for pre-existing differences in cooking familiarity, deficits in attention, memory and executive functioning were found to underlie performance on several HBCT indices (Omissions, Estimations, Goal Achievement and completion time). Such findings highlight that multiple cognitive processes act in concert to support successful performance on complex everyday tasks. Consequently, errors on naturalistic tasks such as the HBCT are unlikely to result from impairment in one specific cognitive domain, but rather arise from deficits in multiple cognitive domains. The pattern of associations between the neuropsychological tests and HBCT also suggests that different cognitive abilities are implicated in particular task requirements. For example, accurate measurement (estimation) was related to the ability to prevent competing internal or external stimuli from disrupting goal-directed behavior. Similarly, participants' capacity to act as if they are alone and inhibit conversation during the task was related to rule following and self-regulation skills.

The finding that various cognitive processes are engaged to support individuals to initiate and carry out complex tasks in their own homes has implications for rehabilitation. In particular, remedial or compensatory approaches targeting a single cognitive domain (e.g., memory training) may not be sufficient to substantially reduce errors on functional tasks. Rather, a focus on preventing or managing errors arising from varied underlying cognitive deficits is likely to be more beneficial. Two contrasting approaches for managing error self-regulation impairments have been evaluated in the rehabilitation literature, namely, errorless learning and metacognitive skills training (Ownsworth et al., 2013).

Errorless learning has mainly been advocated for individuals with severe explicit memory impairment where the aim is to avoid errors from occurring during the initial learning phase to prevent implicit consolidation of error responses (Clare and Jones, 2008). As a bottom-up functional approach, errorless learning is most effective for teaching people task-specific skills such as step-by-step procedures. In a systematic review of the efficacy of cognitive rehabilitation, Cicerone et al. (2011) concluded that errorless learning is recommended for teaching specific information and task-specific procedures to people with severe memory impairment. It was noted that “the presence of severe executive dysfunction may limit effectiveness of this form of memory rehabilitation” (p. 524). In particular, skills taught during training are unlikely to generalize beyond the immediate training context (Clare and Jones, 2008).

In contrast to errorless learning, metacognitive skills training, is a top-down approach designed to enhance individuals' capacity for self-awareness and self-regulation (Ownsworth et al., 2015). Structured learning opportunities on functional tasks are developed for people to make errors, and to become aware of and self-correct their errors. Techniques include role reversal (i.e., the therapist deliberately makes errors for the participant to identify), self-predictions prior to task performance, graded prompts during task performance, and observation and self-reflection on performance through the use of audio-visual recordings and post-task debriefing (Ownsworth et al., 2006; Schmidt et al., 2015). Therefore, irrespective of the cognitive deficits underlying errors, individuals are taught internal self-regulation strategies (i.e., to stop, check and notice errors) and to practice these strategies on different everyday tasks to promote skills generalization.

The decision of whether to use errorless learning or metacognitive skills training to manage errors in daily life depends on goals of the interventions and client characteristics. If errorfree performance on a specific task is a high priority and the individual has severe memory impairment, errorless learning may be most beneficial (Clare and Jones, 2008). Conversely, if the intervention goal is to promote independent self-regulatory skills that can be flexibly applied across everyday tasks to compensate for cognitive impairment more broadly, metacognitive skills training is likely to be more advantageous (Ownsworth et al., 2015).

In summary, the present study identified that deficits in multiple cognitive domains underlie error behavior on a naturalistic task in the home environment. Naturalistic assessment tasks can provide a valuable adjunct to standard neuropsychological tests. More specifically, by providing insight into clients' functional abilities and errors within their own environmental context the HBCT has the potential to guide rehabilitation planning and lifestyle support. Further research is currently underway by the authors to investigate the relationship between performance on the HBCT and indices of real-world functioning, including independent living skills, vocational functioning and care and support needs.

## Author contributions

All authors contributed to the design and conduct of the study and were involved in preparing the manuscript for publication. TO supervised the entire study and the data collection process as conducted by KH and EB. MPC provided training on the administration and scoring of the Cooking Task, and assisted with HBCT scoring. KH prepared the initial draft of the manuscript. JF and JG assisted with participant recruitment. TO and KH conducted the data analysis with the assistance of DS.

### Conflict of interest statement

The authors declare that the research was conducted in the absence of any commercial or financial relationships that could be construed as a potential conflict of interest.
